# Marginal Effects of Multiple Years of Volunteering on Objective and Subjective Measures of Cognition

**DOI:** 10.1093/geroni/igab046.2663

**Published:** 2021-12-17

**Authors:** Yi Wang, Takashi Amano, Huei-wern Shen, Roger Wong

**Affiliations:** 1 University of Iowa, Iowa City, Iowa, United States; 2 Rutgers University-Newark, Newark, New Jersey, United States; 3 University of North Texas, Denton, Texas, United States; 4 SUNY Upstate Medical University, Syracuse, New York, United States

## Abstract

Volunteering is conducive to older Americans’ physical and mental health; however, the effect of volunteering on cognitive health is less studied. Using four waves (2010-2016) of the Health and Retirement Study, this study examined the incremental effect of volunteering engagement on older adults’ cognitive health. We included10,718 cognitively unimpaired, community-dwelling individuals aged 51+ in 2010 and were alive through 2016. Volunteering engagement was measured by the number of times respondents participated in volunteering throughout the four waves. Objective cognition was assessed using the Telephone Interview for Cognitive Status (TICS), a standardized test of cognitive functioning. The TICS score was further categorized into three statuses: “No impairment,” “Cognitive impairment no dementia (CIND),” and “Dementia.” Subjective cognition referred to self-rated memory on a 5-point Likert scale. With sampling weights, ordered logit regression was performed controlling for health-related variables (e.g., health conditions, depression), SES (e.g., income, assets), contextual features (e.g., neighborhood safety, urbanicity), and sociodemographics. The average marginal effects (AMEs) were produced. Results show that more volunteering engagement significantly reduced the likelihood of CIND or dementia (OR=0.88, p<0.001). Specifically, every one-time increase in volunteering increased the probability of remaining cognitively normal by 0.01 (p<0.001), whereas it decreased the probability of CIND by 0.008 (p<0.001) and dementia by 0.001 (p<0.001). For subjective cognition, there was no significant relationship with volunteering. Our findings address gaps in literature by adding evidence of the incremental health benefits of volunteering on cognitive functioning. Differences in the findings for subjective and objective cognition warrant further investigation.

